# Rare Organism Uncommon Disease Case Vignette of Guillain–Barré Syndrome Induced by *Fusobacterium nucleatum* Infection

**DOI:** 10.1155/2021/8816104

**Published:** 2021-03-04

**Authors:** K. Shah, A. Ahmed, K. Kaveh, A. Frugoli, M. Hammoudi

**Affiliations:** ^1^Community Memorial Health System, Graduate Medical Education, Department of Internal Medicine, Ventura, CA, USA; ^2^Western University of the Health Science, Graduate Medical Education, Pomona, CA, USA; ^3^Community Memorial Health System, Graduate Medical Education, Department of Internal Medicine, Pacific Inpatient Physicians, Ventura, CA, USA; ^4^Community Memorial Hospital, Department of Neurology, Ventura, CA, USA

## Abstract

In this case report, we describe an unusual pathogen *F. nucleatum*-induced empyema, followed by the development of Guillain–Barré syndrome (GBS). Although many pathogens have been associated with GBS, this may be one of the few in the literature to describe an association with *F. nucleatum* infection.

## 1. Introduction

Fusobacteria are anaerobic Gram-negative rods with two species causing most human-related illnesses, including *F. nucleatum* and *F. necrophorum*. These organisms are well-documented oral flora species that are capable of a variety of pathologies. *F. nucleatum* is more likely to cause extraoral infections and more commonly affects older individuals with comorbidities [1]. *F. necrophorum* more commonly affects young, healthy adults. It is also associated with rare Lemierre's syndrome characterized by thrombophlebitis of the internal jugular vein and bacteremia following a recent oropharyngeal infection [1].

Moreover, fusobacterial infections can affect the lung and pleura, gastrointestinal organs, and female genital tract. The wide clinical spectrum can range from upper respiratory infections, such as sinusitis or pharyngitis, to abscess formation, septicemia, and septic shock. They account for less than one percent of anaerobic bacteremias, with an incidence of 0.55 per 100,000 people per year [2]. Hematogenous spread has been linked to abscess formations in the liver [2]. Anaerobic bacteria cause less than 1% of middle-ear infection. Rare cases of *Fusobacterium* acute otitis media and mastoiditis have been reported [3]. Fusobacteria infections have low mortality due to low antibiotic resistance.

Guillain–Barré syndrome (GBS) is an uncommon acute autoimmune-mediated polyneuropathy with several variants all characterized by immune-mediated attacks on peripheral nerve components. The annual incidence of GBS is 0.81–1.89 per 100,000 persons worldwide [4]. The syndrome results in an acute and progressive, mostly symmetric, ascending muscle weakness. Symptoms can vary from mild difficulty walking to quadriplegia with bulbar muscles weakness and respiratory failure. Respiratory failure occurs in a quarter of patients who will require ventilator support [5]. 60% of patients are able to walk unaided by 1 year after their illness while the rest are left with various degrees of residual deficits [6].

The underlying pathophysiology is thought to be an immune response secondary to a variety of triggering infections. This immune response cross reacts with peripheral nerve components due to sharing of cross-reactive epitopes. Two-thirds of people with GBS experience a respiratory tract or gastrointestinal infection several weeks before developing symptoms [5]. The most common triggering agents are *Campylobacter jejuni* (in 13% to 39% of cases), followed by cytomegalovirus (5% to 22%), Epstein–Barr virus (1% to 13%), and *Mycoplasma pneumoniae* (5%) [7]. All of these pathogens have carbohydrate sequences (antigens) in common with peripheral nerve tissue [7].

## 2. Case Description

**Figure 1:**
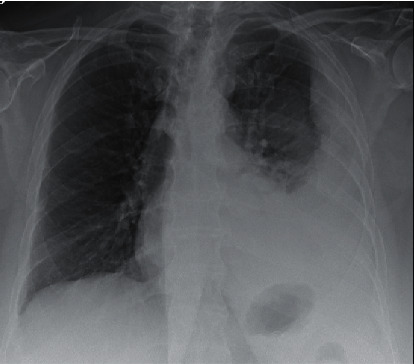
Portable upright chest radiograph, demonstrating loculated left-sided effusion with associated infiltrate and lobar pneumonia.

**Figure 2:**
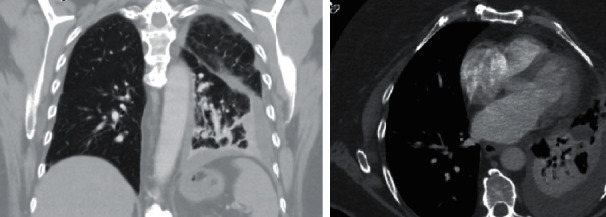
CT chest angiogram demonstrating extensive left lobar pneumonia with surrounding moderate-sized loculated pleural effusion.

A 77-year-old female with spinal stenosis, obstructive sleep apnea, and hyperlipidemia presented to the emergency room with left-sided pleuritic chest pain. Her evaluation demonstrated a left-sided pleural effusion with basilar consolidation (Figure 1). She was started empirically on oral levofloxacin for presumed community-acquired pneumonia with parapneumonic effusion. Her evaluation for the etiology of her pneumonia including respiratory polymerase chain reaction, coccidioidomycosis antibody screen, urinary *Legionella* antigen, urinary Streptococcal antigen, and blood cultures were all unremarkable. She failed empiric treatment and continued to have effusion with loculated appearance on the chest computerized tomography (CT) angiogram (Figure 2). She underwent ultrasound-guided thoracentesis that confirmed exudative pleural effusion. Following thoracentesis, she had ongoing evidence of effusion and underwent CT-guided left-sided 12 French chest tube placement with interventional radiology. Despite conservative management and attempts for drainage, she continued to be symptomatic and had ongoing evidence of effusion. Due to persistent evidence of loculated collections on imaging and minimal output via chest tubes, she underwent thoracoscopic total lung decortication. Cytology from fluid collected from thoracoscopy was consistent with pneumonia and negative for malignant cells. Delayed intraoperative cultures identified growth from pleural fluid with *Fusobacterium nucleatum*. The patient clinically improved during this hospitalization on levofloxacin and was later discharged home after an eleven-day stay.

**Figure 3:**
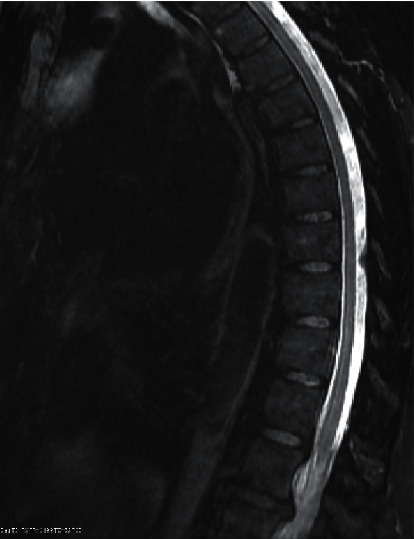
MRI with contrast, demonstrating facet spondylosis and ligamentum flavum thickening at T9-10 with mild central spinal stenosis but no cord compression. Disc degeneration at the thoracolumbar junction.

**Figure 4:**
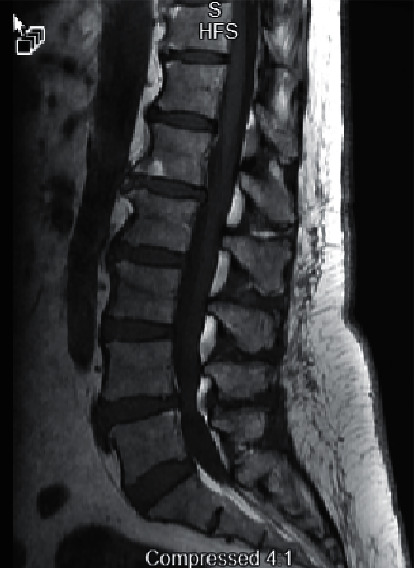
Lumbar MRI with contrast, demonstrating multilevel degenerative changes. There is facet arthropathy and ligamentum flavum hypertrophy. L4-5: there is a diffuse bulge slightly larger on the right. There is severe central canal stenosis. There is moderate right and mild-to-moderate left neural foraminal stenosis. L5-S1: there is a mild diffuse bulge slightly larger on the left with a tiny left foraminal annular tear. There is severe left and moderate right neural foraminal stenosis. L1-2: there is diffuse bulge and a superimposed central protrusion. There is mild-to-moderate central spinal canal stenosis.

She completed her antibiotic course and was recovering well up until 3 weeks after the initial pneumonia presentation when she woke up with intractable back pain and associated leg weakness. She returned to the emergency room for evaluation of her symptoms that also included feet numbness and tingling, vague pain in her legs, especially her thighs, and finally, tightness and tingling sensation in her hands and wrists. Emergency room evaluation included thoracic and lumbar spine MRI. Thoracic MRI imaging identified facet spondylosis and ligamentum flavum thickening at T9-10 with mild central spinal stenosis (Figure 3). Lumbar MRI identified L4-L5 with diffuse bulge slightly larger on the right resulting in severe central canal stenosis (Figure 4). There is moderate right and mild-to-moderate left neural foraminal stenosis. She was started on steroids and gabapentin in addition to pain medications. Her pain, numbness, and lower extremity weakness continued, and neurology was consulted. Her examination was positive for mild symmetric weakness of small hand muscles 4+/4+, hip flexion 4−/4−, hip adductors 4/4, hip abductors 4+/4+, big toe extensors 4−/4−, quadriceps 5−/5−, foot dorsiflexion 5−/5−, and foot eversion 4+/4+. Sensation was normal to pinprick and light touch but decreased to vibration in the lower extremities up to the hips. She had global areflexia. Additional testing included MRI of the cervical spine that did not demonstrate any significant disease. Her best working diagnosis was Guillain–Barré syndrome. This was further supported by CSF analysis showing WBC of 0 and protein 199. At that time, the patient reported minor improvement over the preceding 2 days. Based on minor deficits and stability of her symptoms, she was discharged to an acute rehabilitation facility on a steroid taper. She was able to recover, and on her 3-month follow-up, her examination showed normal strength to all muscle groups and recovery of deep tendon reflexes.

## 3. Discussion

Although *F. nucleatum* rarely causes severe infections, it is associated with pleuropulmonary disease. In this case, *F. nucleatum* is the likely etiology for complicated pneumonia with empyema as it was the only organism identified from the pleural fluid. We postulate that this organism may have been the inciting etiology that led to the development of Guillain–Barré syndrome. As with the multiple organisms associated with GBS, it is impossible to prove the cause and effect, but the timing and clinical course are suggestive. The patient's evaluation did not identify any lesion on neuroimaging that would correlate with all of her symptoms. Additionally, her lumbar puncture with elevated protein and normal glucose and cell count which is pathognomonic for GBS. This albuminocytologic dissociation is usually evident within 1–3 weeks of contracting Guillain–Barré which is fitting for her timeline of recovery from *F. nucleatum*. Her symptoms were also characteristic of Guillain–Barré syndrome with bilateral lower extremity weakness, numbness, and tingling sensation. Her lower back pain initially misled her care providers, but still represents a less common symptom for GBS [8]. The severity of the patient's pulmonary illness during her prior admission and the rapid onset of her bilateral lower extremity pain and numbness within 3 weeks of discharge correlate with the usual progression of Guillain–Barré. It should be noted that one of the limitations is lack of electrophysiologic studies.

Treatment with IVIG or plasmapheresis is usually considered for nonambulatory patients within 4 weeks of symptom onset. The beneficial effects of both forms of treatment are believed to be equivalent, and the decision to use one treatment versus the other is dependent on availability, patient preference, and contraindications. The primary adverse reactions from plasmapheresis are hypotension and sepsis. Side effects for IVIG include rash, acute renal failure, and anaphylaxis in patients with IgA deficiency. IVIG is often preferred because the treatment duration is 5 days whereas plasmapheresis is usually given as 4 to 6 treatments over 10 days. Plasmapheresis is also most effective when initiated within 7 days of symptom onset. The patient was ultimately not treated with IVIG as the patient's symptoms had plateaued during her hospital course, with significant improvement in her pain and improved strength. She was discharged to a skilled nursing facility for supportive treatment due to ongoing debility and weakness.

## 4. Conclusions

In this case vignette, we describe a clinical presentation of an uncommon infection with *Fusobacterium nucleatum* resulting in empyema in an immunocompetent host, followed by delayed development of mild Guillain–Barré syndrome that improved with supportive measures without use of plasmapheresis or IVIG. Additionally, we provide theory that the organism, *F. nucleatum*, is the likely culprit for induction of GBS for this patient. This case provides an interesting link between two uncommon disease processes and suggests new information that *F. nucleatum* may be associated with GBS.

## Data Availability

Data are disclosed in the manuscript and are limited in this single patient case report.

## References

[1] Kevin A., Laupland K., Leal J., Lloyd T., Gregson D. (2013). Incidence, risk factors, and outcomes of fusobacterium species bacteremia. *BMC Infectious Diseases*.

[2] Jayasimhan D., Wu L., Huggan P. (2017). Fusobacterial liver abscess: a case report and review of the literature. *BMC Infectious Diseases*.

[3] Arane K., Goldman R. D. (2016). Fusobacterium infections in children. *Canadian family physician Medecin de famille canadien*.

[4] Baughman A. L., Wise M., Morgan O. W. (2011). Population incidence of guillain-barré syndrome: a systematic review and meta-analysis. *Neuroepidemiology*.

[5] Willison H., Jacobs B., Van Doorn P. (2016). Guillain-Barré syndrome. *The Lancet*.

[6] Winer J. B. (2014). An update in guillain-barré syndrome. *Autoimmune Diseases*.

[7] Yu R. K., Usuki S., Ariga T. (2006). Ganglioside molecular mimicry and its pathological roles in guillain-barré syndrome and related diseases. *Infection and Immunity*.

[8] Yao S., Chen H., Zhang Q. (2018). Pain during the acute phase of guillain-barré syndrome. *Medicine*.

